# Twin pregnancies are risk factors for both early- and late-onset hypertensive disorders of pregnancy: the Japan Environment and Children’s study

**DOI:** 10.1038/s41440-025-02502-7

**Published:** 2026-01-09

**Authors:** Kazuma Tagami, Noriyuki Iwama, Hirotaka Hamada, Hasumi Tomita, Natsumi Kumagai, Hongxin Wang, Seiya Izumi, Zen Watanabe, Mami Ishikuro, Taku Obara, Hirohito Metoki, Yuichiro Miura, Chiharu Ota, Shinichi Kuriyama, Takahiro Arima, Nobuo Yaegashi, Masatoshi Saito, The Japan Environment and Children’s Study Group

**Affiliations:** 1 Department of Obstetrics and Gynecology, Tohoku University Graduate School of Medicine, Sendai, Miyagi Japan; 2 Center for Maternal and Perinatal Medicine, Tohoku University Hospital, Sendai, Miyagi Japan; 3 Division of Molecular Epidemiology, Department of Preventive Medicine and Epidemiology, Tohoku Medical Megabank Organization, Tohoku University, Sendai, Miyagi Japan; 4 Division of Molecular Epidemiology, Tohoku University Graduate School of Medicine, Sendai, Miyagi Japan; 5 Division of Public Health, Hygiene and Epidemiology, Tohoku Medical Pharmaceutical University, Sendai, Miyagi Japan; 6 Tohoku Medical Megabank Organization, Tohoku University, Sendai, Miyagi Japan; 7 Environment and Genome Research Center, Tohoku University Graduate School of Medicine, Sendai, Miyagi Japan; 8 Department of Paediatrics, Tohoku University Graduate School of Medicine, Sendai, Miyagi Japan; 9 International Research Institute of Disaster Science, Tohoku University, Sendai, Miyagi Japan; 10 Department of Maternal and Fetal Therapeutics, Tohoku University Graduate School of Medicine, Sendai, Miyagi Japan

**Keywords:** Dichorionic diamniotic twin, Monochorionic diamniotic twin, Hypertensive disorders of pregnancy, Morning hypertension

## Abstract

This study investigated the associations of twin pregnancies with early-onset (EO)- and late-onset (LO)-hypertensive disorders of pregnancy (HDP). Totally, 86,717 pregnant women were included in a prospective birth cohort study. The associations of dichorionic diamniotic (DD)- and monochorionic diamniotic (MD)-twin pregnancies with EO-HDP (Diagnosed from 20 to <34 weeks of gestation) and LO-HDP (Diagnosed at ≥34 weeks of gestation) were analyzed using a multinomial logistic regression model. Compared with singleton pregnancies, both DD- and MD-twin pregnancies had significantly higher odds for EO- and LO-HDP. The adjusted odds ratios (aORs) for EO-HDP were 2.05 (95% confidence interval [Cl]: 1.51–2.78) in DD-twin pregnancies and 2.80 (95% Cl: 2.01–3.90) in MD-twin pregnancies, respectively. Also, the aORs for LO-HDP were 1.32 (95% CI: 1.03–1.69) in DD-twin pregnancies and 1.64 (95% Cl: 1.24–2.17) in MD-twin pregnancies, respectively. Although no statistically significant differences were observed, MD-twin pregnancies tended to have higher odds for both EO- and LO-onset HDP compared with DD-twin pregnancies. In conclusion, both DD- and MD-twin pregnancies are risk factors for the development of EO- and LO-HDP.

**Figure:**
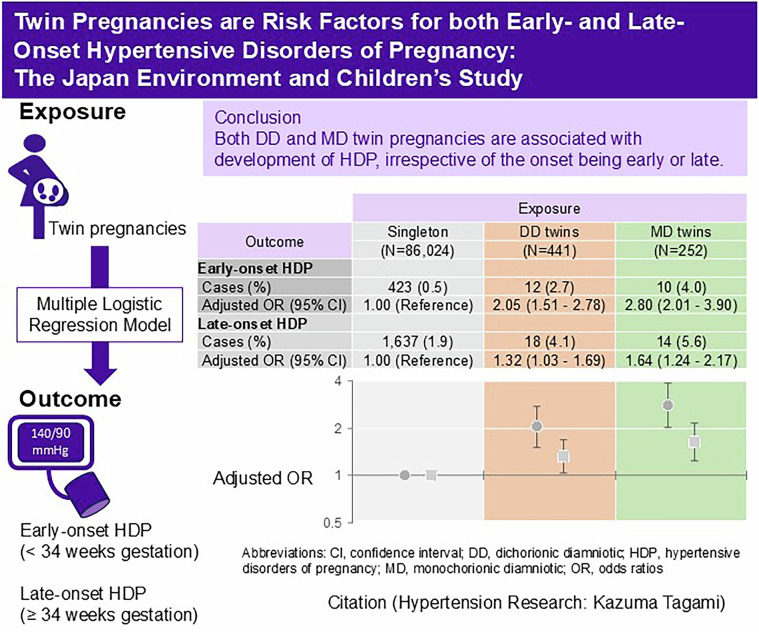
We showed that both dichorionic diamniotic and monochorionic diamniotic twin pregnancies are risk factors for the development of early-onset and late-onset hypertensive disorders of pregnancy

We showed that both dichorionic diamniotic and monochorionic diamniotic twin pregnancies are risk factors for the development of early-onset and late-onset hypertensive disorders of pregnancy

## Abstract (web summary)

We showed that both dichorionic diamniotic and monochorionic diamniotic twin pregnancies are risk factors for the development of early-onset and late-onset hypertensive disorders of pregnancy

## Introduction

The global prevalence of pregnant women with twin pregnancies has been increasing [1, 2]. In Japan, twin pregnancies accounted for 1% of all pregnancies in 2019 [3]. Twin pregnancies involve greater alterations for women in cardiac output, systemic vascular resistance, and blood volume than singleton pregnancies [4–6]. Moreover, compared to singleton pregnancies, twin pregnancies are associated with an increased risk of perinatal complications, including preterm birth, maternal renal dysfunction, postpartum hemorrhage, postpartum depression, fetal structural abnormalities, and aneuploidy [3, 7–9].

Based on the number of chorions and amnions, twin pregnancies are classified into dichorionic diamniotic (DD)-, monochorionic diamniotic (MD)-, and monochorionic monoamniotic (MM)-twin pregnancies. Compared with DD-, MD-twin pregnancies are associated with a higher incidence of preterm birth and poorer neurological outcomes in the offspring [10]. Additionally, because they share a common placenta, MD- and MM-twin pregnancies have specific risks of complications, such as twin-to-twin transfusion syndrome, which contributes to adverse perinatal prognosis [11]. Risk factors for twin pregnancies include assisted reproductive technology (ART), advanced maternal age, higher parity, and smoking [1]. Moreover, ART and advanced maternal age have been associated with an increased proportion of DD- compared to MD-twin pregnancies [1]. To investigate the association between twin pregnancies and perinatal complications, the effects of these background factors need to be considered.

Hypertensive disorders of pregnancy (HDP) are common complications associated with adverse perinatal outcomes, including stillbirth, preterm birth, perinatal cardiomyopathy, and postpartum hemorrhage [12, 13]. Furthermore, women who have developed HDP are also at an increased risk of non-communicable diseases later in life [13, 14]. Additionally, offspring born to mothers with HDP are at an increased risk of developing hypertension, lifestyle-related diseases, and neurological disorders later in life [15]. Several risk factors for HDP have been reported, including ART, obesity, low maternal birth weight (i.e., maternal birth weight <2500 g), primiparity, diabetes mellitus, and a family history of hypertension [13, 16, 17]. Moreover, compared to singleton pregnancies, twin pregnancies are associated with higher blood pressure [18] and are significant risk factors for HDP [13, 16, 19, 20]. However, the definitive etiology of twin pregnancies and HDP, especially considering the subtypes described below, remains unknown.

HDP are divided into two subtypes based on the gestational age at diagnosis, early-onset (EO)-HDP (diagnosed from 20 to <34 weeks of gestation) and late onset (LO)-HDP (diagnosed at ≥34 weeks of gestation) [21]. Pathology and perinatal prognosis have been reported to differ between EO- and LO-HDP [21]. EO-HDP is associated with a higher incidence of adverse perinatal outcomes, preterm birth, EO-FGR, recurrence of HDP in subsequent pregnancies, and maternal cardiovascular disease after childbirth, compared to LO-HDP [21–24]. Additionally, blood pressure levels during gestation differ between DD- and MD-twin pregnancies [18]. Therefore, the risk of HDP onset may differ according to the timing of DD and MD pregnancies.

In Japan, the proportion of pregnancies resulting from ART and advanced maternal age is increasing, and there are concerns that the proportion of twin pregnancies and HDP will further increase in the future [25]. Therefore, it is important to investigate the association between twin pregnancies and HDP. Although only a few studies have reported an association between twin pregnancies and HDP or PE classified according to gestational age at diagnosis [26, 27], these studies have no Japanese population. The incidence and risk factors for HDP, as well as the proportion of twin pregnancies, are known to vary by ethnicity, reflecting differences in genetic background, environmental exposures, and socioeconomic conditions [2, 28, 29]. For example, the association between smoking and HDP varies by ethnicity. Although a lower risk was observed among non-Hispanic white and non-Hispanic American Indian pregnant women who smoked, a higher risk was observed among non-Hispanic Black, non-Hispanic Asian, and Hispanic women [30]. Similarly, a higher risk associated with smoking during pregnancy has also been observed among Japanese women [31]. Hence, these findings suggest that the association between twin pregnancies and the risk of HDP, classified according to gestational age at diagnosis, may differ between the Japanese population and populations in other countries. This study aimed to investigate the association between twin pregnancies and HDP by classifying twin pregnancies into DD and MD groups, and further categorizing HDP into EO and LO groups.

Point of view

- Clinical relevance
  Both monochorionic diamniotic (MD) and dichorionic diamniotic (DD) twin pregnancies were identified as risk factors for early-onset (EO) and late-onset (LO) hypertensive disorders of pregnancy (HDP). Proactive risk assessment and blood pressure management are essential, particularly during the first trimester. In MD-twin pregnancies, where the risk of HDP onset is higher, thorough risk evaluation and frequent monitoring are especially critical.
- Future directions
  Future longitudinal studies incorporating biomarkers, such as the soluble fms-like tyrosine kinase-1 (sFlt-1)/placental growth factor (PlGF) ratio and renin–angiotensin system (RAS)-related factors, along with ambulatory blood pressure monitoring (ABPM), are warranted. Such approaches are expected to advance the development of HDP prevention strategies specifically tailored to twin pregnancies.
- Considerations for the Asian population
  This study is the first to clarify the association between chorionicity in twin pregnancies and the timing of HDP onset in a Japanese cohort. These findings are expected to inform the development of prevention and management guidelines for HDP that consider the genetic background and lifestyle characteristics of Asian populations, including Japanese women.

## Materials and methods

### Study design

This study collected data from the Japan Environment and Children’s Study (JECS), an ongoing prospective birth cohort study conducted in Japan. The details of the JECS study design have been previously described [32, 33]. The study was conducted in accordance with the provisions of the Declaration of Helsinki in 1995 (as revised in Helsinki, Finland, October 2024). Written informed consent was obtained from all study participants in the JECS. The JECS protocol was reviewed and approved by the Ministry of the Environment’s Institutional Review Board on Epidemiological Studies and Ethics Committees of all participating institutions. The JECS aims to investigate environmental factors that affect children’s health and development. Participants included pregnant women and their partners recruited between January 2011 and March 2014 from 15 Regional Centres: Hokkaido, Miyagi, Fukushima, Chiba, Kanagawa, Koshin, Toyama, Aichi, Kyoto, Osaka, Hyogo, Tottori, Kochi, Fukuoka, and South Kyushu/Okinawa. This study used the “jecs-ta-20190930” dataset released by the Programme Office in October 2019. In the JECS, questionnaires including maternal information were administered in the first trimester (namely, MT1), second or third trimester (namely, MT2), and six months after childbirth (namely, C6m), respectively. This study was funded by the Ministry of Environment, Japan.

### Exposure (the number of fetuses)

The number of fetuses was transcribed from the medical records. Pregnant women were classified as singleton or twin pregnancies based on the number of fetuses. For twin pregnancies, the classification was further divided into DD-, MD-, and MM-twin pregnancies based on chorionicity and amnionicity. Because of the small sample size, this study excluded triplet and MM-twin pregnancies.

### Outcome (EO- and LO-HDP)

HDP was transcribed from the medical records. Based on the Japanese Society of Hypertension in Pregnancy guidelines, HDP was defined as a systolic blood pressure (SBP) of ≥140 mmHg and/or diastolic blood pressure (DBP) of ≥90 mmHg [21]. Because this study focused on new-onset HDP occurring at ≥20 weeks of gestation, pregnant women with chronic hypertension were excluded. Moreover, HDP was further divided into two subtypes based on the gestational age upon diagnosis: EO-HDP (diagnosed from 20 to <34 weeks of gestation) and LO-HDP (diagnosed at ≥34 weeks of gestation) [21].

### Data collection of other variables

Data regarding maternal height, pre-pregnancy body weight (BW), parity, conception methods, and gestational diabetes mellitus (GDM), SBP, and DBP were transcribed from the medical records. If medical record transcription was unavailable, maternal height and pre-pregnancy BW were obtained from the MT1 questionnaire. Pre-pregnancy body mass index (BMI) was calculated as follows: pre-pregnancy BMI = BW in kg / (height in m)². Data regarding maternal age, smoking status, alcohol drinking status, marital status, history of kidney disease, mental disorders, type 1 diabetes mellitus, and type 2 diabetes mellitus were collected from the MT1 questionnaire. Data regarding the highest maternal education level and annual household income (million, Japanese yen) were obtained from the MT2 questionnaire. Maternal birth weight was obtained from the C6m questionnaire. Marital status included married, never married, and divorced or widowed. Kidney diseases included chronic nephritis and nephrotic syndrome. Mental disorders included depression, dysautonomia, anxiety, and schizophrenia. Smoking status included “Never”, “Previously did, but quit before realizing current pregnancy”, “Previously did, but quit after realizing current pregnancy,” and “Currently smoking.” Alcohol drinking status included “Never”, “Quit drinking before,” and “Continued drinking”. Parity was classified into the primipara and multipara. The conception methods included spontaneous pregnancy, non-ART (Ovulation induction and artificial insemination by husband), and ART. The highest level of maternal education was categorized into two groups: <13 years (junior high school and high school) and ≥13 years (technical college, vocational college, junior college, university, and graduate school [master’s and doctoral]). Annual household income included <4 and ≥4 million Japanese yen. Mean arterial pressure (MAP) level during early gestation (i.e., at <16 weeks of gestation) was calculated as follows: DBP + (SBP − DBP)/3. An MAP level < −5 SD or > +5 SD was considered clinically implausible and treated as missing data. Regions where the Regional Centres exist include Hokkaido, Tohoku, Kanto, Chubu, Kinki, Chugoku, Shikoku, and Kyushu-Okinawa.

### Statistical analysis

Categorical variables of the study participants’ characteristics were expressed as numbers (percentages). Continuous variables are expressed as either mean (SD) or median (interquartile range), depending on the data distribution. A multinomial logistic regression model was used to investigate the association between DD- and MD-twin pregnancies and EO- and LO-HDP. Singleton pregnancies were used as a reference. Model 1 was the crude model, and models 2 and 3 were the adjusted models. Model 2 was adjusted for maternal birth weight, maternal age, pre-pregnancy BMI, parity, conception method (spontaneous pregnancy, non-ART, and ART), maternal highest education level, annual household income, smoking status, alcohol status, history of kidney disease, and diabetes mellitus (including type 1 and type 2) [13, 16, 17]. In Model 3, the MAP level during early gestation, as a possible intervening variable, was adjusted for in addition to Model 2. After confirming no strong multicollinearity among the covariates, multiple imputations using a fully conditional specification (FCS) were conducted to deal with the missing data of several covariates in Models 2 and 3 [34]. Ten datasets were created using FCS, and each dataset was analyzed using the same analytical model. Finally, the results of these datasets were combined based on Rubin’s rule and reported as the results of Models 2 and 3. Statistical analyses were performed using SAS software (version 9.4; SAS Institute Inc., Cary, North Carolina, USA) and R (version 4.1.2) [35].

As an additional analysis, a multinomial logistic regression model was applied to compare the strength of the associations between EO- and LO-HDP in DD- and MD-twin pregnancies, with DD-twin pregnancies as the reference group.

## Results

### Study participants

**Fig. 1:**
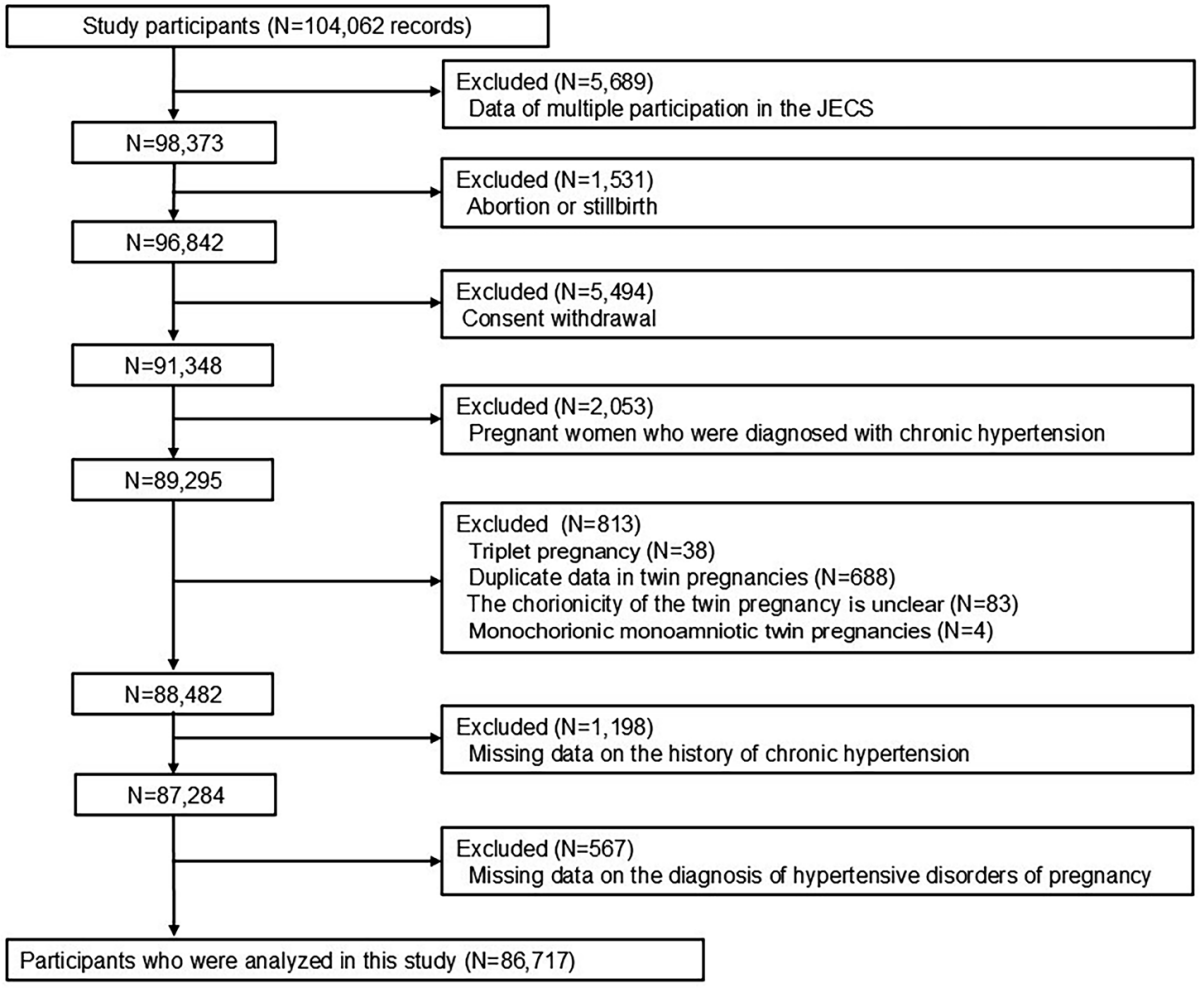
Flowchart of study participants selection

The flowchart of the participant selection is shown in Fig. 1. The JECS included 104,062 records. We excluded data of multiple participation in the JECS (*N* = 5689), pregnant women who had an abortion or stillbirth (*N* = 1531), pregnant women with consent withdrawal (*N* = 5494), pregnant women who were diagnosed with chronic hypertension (*N* = 2053), pregnant women with triplet pregnancy (*N* = 38), duplicate data in twin pregnancies (*N* = 688), pregnant women with twin pregnancy whose the chorionicity is unclear (*N* = 83), pregnant women with MM-twin pregnancies (*N* = 4), pregnant women with missing data on the history of chronic hypertension (*N* = 1198), and pregnant women with missing data on the diagnosis of HDP (*N* = 567). Finally, 86,717 pregnant women were included in this study.

### Characteristics of study participants

Table 1 shows the maternal and neonatal characteristics of study participants; Among them, the numbers (percentages) were 86,024 (99.2%) in singleton, 441 (0.5%) in DD-, and 252 (0.3%) in MD-twin pregnancies, respectively. The numbers (percentages) of EO- and LO-HDP were 445 (0.5%) and 1669 (1.9%), respectively. The proportions of EO- and LO-HDP were higher in twin pregnancies than in singleton pregnancies. In DD-twin pregnancies, the numbers (percentages) of EO- and LO-HDP were 12 (2.7%) and 18 (4.1%), respectively. Furthermore, in MD-twin pregnancies, the numbers (percentages) of EO- and LO-HDP were 10 (4.0%) and 14 (5.6%), respectively. The number (percentages) of conceptions via ART was 71 (16.1%) for DD- and 21 (8.3%) for MD-twin pregnancies. The mean (SD) gestational age at delivery in DD- and MD-twin pregnancies was 36.5 (2.0) and 35.9 (2.4) weeks of gestation, respectively.

**Table 1:** The maternal and neonatal characteristics of study participants

Variables	All participants (*N* = 86,717) ^1^	Singleton pregnancies (*N* = 86,024) ^1^	Twin pregnancies	
			DD-twin pregnancies (*N* = 441) ^1^	MD-twin pregnancies (*N* = 252) ^1^
**Maternal age, N (%)**	30.8 (5.0)	30.8 (5.0)	31.9 (4.9)	30.4 (4.8)
<25 years	9672 (11.2)	9616 (11,2)	31 (7.0)	25 (9,9)
25–29.9 years	25,449 (29.3)	25,242 (29.3)	115 (26.1)	92 (36.5)
30–34.9 years	30,301 (34.9)	30,064 (34.9)	150 (34.0)	87 (34.5)
35–39.9 years	18,146 (20.9)	17,989 (20.9)	119 (27.0)	38 (15.1)
≥40 years	3145 (3.6)	3109 (3.6)	26 (5.9)	10 (4.0)
Missing	4 (0.0)	4 (0.0)	0 (0.0)	0 (0.0)
**Maternal birth weight, N (%)**				
<2500 g	3814 (4.4)	3783 (4.4)	21 (4.8)	10 (4.0)
2500–3999 g	72,370 (83.5)	71,794 (83.5)	366 (83.0)	210 (83.3)
≥4000 g	1780 (2.1)	1765 (2.1)	10 (2.3)	5 (2.0)
Missing	8753 (10.1)	8682 (10.1)	44 (10.0)	27 (10.7)
**Maternal height, cm**	158.1 (5.3)	158.1 (5.3)	158.8 (5.3)	157.8 (5.3)
**Pre-pregnancy BMI kg/m**^**2**^**, N (%)**	21.1 (3.1)	21.1 (3.1)	21.4 (3.4)	21.0 (3.4)
Underweight (<18.5 kg/m^2^)	14,225 (16.4)	14,104 (16.4)	71 (16.1)	50 (19.8)
Normal range (18.5–24.9 kg/m^2^)	63,907 (73.7)	63,408 (73.7)	321 (72.8)	178 (70.6)
Overweight/Obesity (≥25.0 kg/m^2^)	8551 (9.9)	8478 (9.9)	49 (11.1)	24 (9.5)
Missing	34 (0.0)	34 (0.0)	0 (0.0)	0 (0.0)
**Parity, N (%)**				
Primipara	48,528 (56.0)	48,199 (56.0)	206 (46.7)	123 (48.8)
Multipara	36,089 (41.6)	35,735 (41.5)	226 (51.2)	128 (50.8)
Missing	2100 (2.4)	2090 (2.4)	9 (2.0)	1 (0.4)
**Conception method, N (%)**				
Spontaneous pregnancy	80,430 (92.7)	79,970 (93.0)	237 (53.7)	223 (88.5)
Non-ART	3284 (3.8)	3145 (3.7)	133 (30.2)	6 (2.4)
ART	2636 (3.0)	2544 (3.0)	71 (16.1)	21 (8.3)
Missing	367 (0.4)	365 (0.4)	0 (0.0)	2 (0.8)
**MAP level during early gestation, N (%)**	79.1 (9.0)	79.1 (9.0)	81.1 (8.9)	81.7 (9.0)
**SLE and/or APS**	200 (0.2)	198 (0.2)	1 (0.2)	1 (0.4)
**The history of kidney disease, N (%)**	364 (0.4)	360 (0.4)	3 (0.7)	1 (0.4)
**The history of mental disorders, N (%)**	6771 (7.8)	6731 (7.8)	26 (5.9)	14 (5.6)
**Type 1 diabetes mellitus**	60 (0.1)	60 (0.1)	0 (0.0)	0 (0.0)
**Type 2 diabetes mellitus**	97 (0.1)	96 (0.1)	1 (0.2)	0 (0.0)
**Smoking status, N (%)**				
Never	50,467 (58.2)	50,053 (58.2)	260 (59.0)	154 (61.1)
Previously did, but quit before realizing current pregnancy	20,039 (23.1)	19,878 (23.1)	108 (24.5)	53 (21.0)
Previously did, but quit after realizing current pregnancy	11,575 (13.3)	11,477 (13.3)	62 (14.1)	36 (14.3)
Currently smoking	4005 (4.6)	3985 (4.6)	11 (2.5)	9 (3.6)
Missing	631 (0.7)	631 (0.7)	0 (0.0)	0 (0.0)
**Alcohol drinking status, N (%)**				
Never	29,698 (34.2)	29,477 (34.3)	146 (33.1)	75 (29.8)
Quit drinking before	47,874 (55.2)	47,469 (55.2)	246 (55.8)	159 (63.1)
Continued drinking	8756 (10.1)	8689 (10.1)	49 (11.1)	18 (7.1)
Missing	389 (0.4)	389 (0.5)	0 (0.0)	0 (0.0)
**Highest maternal education level, N (%)**				
<13 years	30,446 (35.1)	30,230 (35.1)	137 (31.1)	79 (31.3)
≥13 years	55,130 (63.6)	54,667 (63.5)	295 (66.9)	168 (66.7)
Missing	1141 (1.3)	1127 (1.3)	9 (2.0)	5 (2.0)
**Annual household income (million, Japanese Yen), N (%)**				
<4	31,841 (36.7)	31,603 (36.7)	145 (32.9)	93 (36.9)
≥4	39,374 (45.4)	39,068 (45.4)	196 (44.4)	110 (43.7)
Missing	15,502 (17.9)	15,353 (17.8)	100 (22.7)	49 (19.4)
**Marital status, N (%)**				
Married	82,439 (95.1)	81,772 (95.1)	431 (97.7)	236 (93.7)
Unmarried	3148 (3.6)	3133 (3.6)	6 (1.4)	9 (3.6)
Divorced or widowed	746 (0.9)	738 (0.9)	3 (0.7)	5 (2.0)
Missing	384 (0.4)	381 (0.4)	1 (0.2)	2 (0.8)
**New-onset HDP, N (%)**				
Early-onset HDP	445 (0.5)	423 (0.5)	12 (2.7)	10 (4.0)
Late-onset HDP	1669 (1.9)	1637 (1.9)	18 (4.1)	14 (5.6)
**GDM, N (%)**	2268 (2.6)	2239 (2.6)	15 (3.4)	14 (5.6)
**Gestational weeks at delivery, weeks**	39.2 (1.6)	39.3 (1.5)	36.5 (2.0)	35.9 (2.4)
**Regions where**				
**Regional Centres exist, N (%)**				
Hokkaido	6852 (7.9)	6824 (7.9)	10 (2.3)	18 (7.1)
Tohoku	19,097 (22.0)	18,930 (22.0)	103 (23.4)	64 (25.4)
Kanto	10,266 (11.8)	10,219 (11.9)	35 (7.9)	12 (4.8)
Chubu	15,668 (18.1)	15,545 (18.1)	80 (18.1)	43 (17.1)
Kinki	14,695 (16.9)	14,538 (16.9)	104 (23.6)	53 (21.0)
Chugoku	2622 (3.0)	2593 (3.0)	21 (4.8)	8 (3.2)
Shikoku	5975 (6.9)	5934 (6.9)	23 (5.2)	18 (7.1)
Kyushu-Okinawa	11,542 (13.3)	11,441 (13.3)	65 (14.7)	36 (14.3)

^1^Continuous variables are expressed as mean (SD). Categorical variables are expressed as *N* (%)

*APS* antiphospholipid antibody syndrome, *ART* assisted reproductive technology, *BMI* body mass index, *DD* dichorionic diamniotic, *GDM* gestational diabetes mellitus, *HDP* hypertensive disorders of pregnancy, *MAP* mean arterial pressure, *MD* monochorionic diamniotic, *SD* standard deviation, *SLE* systemic lupus erythematosus

### The associations of DD- and MD-twin pregnancies with EO- and LO-HDP

**Fig. 2:**
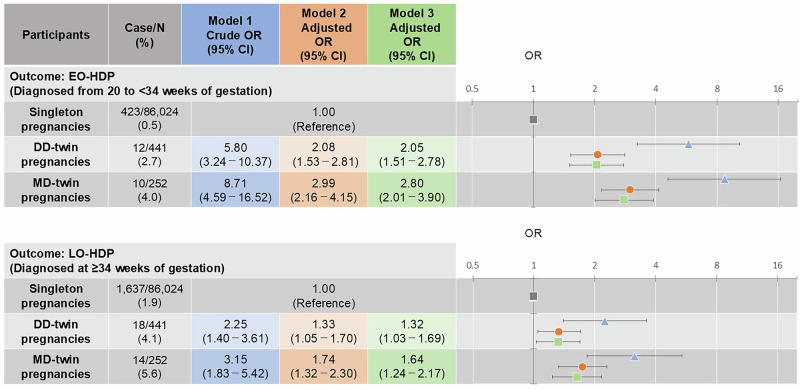
The associations of DD- and MD-twin pregnancies with EO- and LO-HDP. Model 1: Crude model. Model 2: Adjusted for maternal birth weight, maternal age, pre-pregnancy BMI, parity, conception method (spontaneous pregnancy, non-ART, and ART), maternal highest education level, annual household income, smoking status, alcohol status, the history of kidney disease, and diabetes mellitus (including type 1 and type 2). Model 3: Adjusted for mean arterial blood pressure during early gestation in addition to Model 2. ART assisted reproductive technology, BMI body mass index, CI confidence interval, DD dichorionic diamniotic, EO early onset, HDP hypertensive disorders of pregnancy, LO late onset, MD monochorionic diamniotic, OR odds ratio

Figure 2 shows the associations of DD- and MD-twin pregnancies with EO- and LO-HDP. Compared with singleton pregnancies, DD- and MD-twin pregnancies had significantly higher odds of EO-onset HDP in model 2 (adjusted odds ratio [aOR]: 2.08, 95% confidence interval [Cl]: 1.53–2.81 in DD-twin pregnancies, and aOR: 2.99, 95% Cl:2.16–4.15 in MD-twin pregnancies, respectively) and model 3 (aOR: 2.05, 95% Cl:1.51–2.78 in DD-twin pregnancies, and aOR: 2.80, 95% Cl:2.01–3.90 in MD-twin pregnancies, respectively). Similarly, both DD- and MD-twin pregnancies had significantly higher odds of LO-HDP in model 2 (aOR: 1.33, 95% CI: 1.05–1.70 in DD-twin pregnancies, and aOR: 1.74, 95% CI: 1.32–2.30 in MD-twin pregnancies, respectively) and model 3 (aOR: 1.32, 95% CI: 1.03–1.69 in DD-twin pregnancies, and aOR: 1.64, 95% CI: 1.24–2.17 in MD-twin pregnancies, respectively).

Supplementary Fig. 1 also shows the differences in the risk of EO- and LO-HDP between DD- and MD-twin pregnancies. Although no statistically significant differences were observed, MD-twin pregnancies tended to have higher odds of EO- and LO-HDP than DD-twin pregnancies. The detailed association is shown in the Supplementary Materials.

## Discussion

Based on current knowledge, this is the first study to investigate the association between twin pregnancies categorized by chorionicity (i.e., MD- and DD-twin) and HDP categorized by gestational age at diagnosis (i.e., EO- and LO-HDP) in the Japanese population. The large sample size and the adjustment for multiple confounding factors, including socioeconomic factors and MAP levels during early pregnancy, enabled the evaluation of differences in the risk of HDP in twin pregnancies according to chorionicity and onset timing, thereby allowing for a more detailed inference of the underlying pathophysiological differences. This study demonstrated an association between twin pregnancies and a higher risk of developing EO- and LO-HDP. Moreover, the association between twin pregnancies and EO-HDP was stronger than that between twin pregnancies and LO-HDP. Although not statistically significant, the aOR for HDP tended to be higher in MD- compared to DD-twin pregnancies, regardless of gestational age at diagnosis.

Using singleton pregnancies as the reference, previous studies investigating the association between twin pregnancies—without stratification by chorionicity—and HDP have reported an aOR of 3.2 and an adjusted relative risk (aRR) of 1.85 [19, 20]. The aOR observed in the present study is comparable to those reported in previous studies, demonstrating consistency. Although several studies have investigated the relationship between chorionicity and the risk of PE, no clear consensus has been established [36]. Consistent with the findings of the present study, multiple studies have reported no statistically significant difference in the prevalence of PE between monochorionic and DD-twin pregnancies [37–40]. While Campbell et al. have suggested a higher risk of PE in MD- compared to DD-twin pregnancies [41], others have suggested the opposite [27, 42]. Importantly, these studies did not include Japanese populations, and to date, no previous study has specifically examined this issue in a Japanese cohort. Francisco et al. also reported no statistically significant difference in the overall prevalence of PE between monochorionic and DD-twin pregnancies [43]. However, when singleton pregnancies were used as the reference group, the relative risk of PE was 3.5 in DD- and 2.6 in monochorionic twin pregnancies [43]. In contrast, for preterm PE, the relative risk was 8.7 in DD- and 9.1 in monochorionic twin pregnancies [43]. This trend toward an increased risk of PE at earlier gestational ages in both MD- and DD-twin pregnancies is consistent with the findings of our study. Furthermore, Robillard et al. reported that the aOR of EO-HDP was 3.7 (95% Cl: 2.1–6.4) in DD- and 4.0 (95% Cl: 1.6–9.9) in MD, compared to singleton pregnancies, which was comparable to the effect sizes observed in the present study [26]. However, for LO-HDP, the aOR was 2.1 (95% Cl: 1.3–3.4) in DD- and 1.4 (95% Cl: 0.5–4.6) in MD-twin pregnancies, with the result for MD-twin pregnancies differing from that of the present study [26]. This previous study was conducted in a single-center study in France, and the adjustment factors did not include socioeconomic factors and MAP, which were included in the present study. This study differs from previous studies in several key aspects, not only in the classification of HDP based on gestational age (EO- and LO-) and chorionicity, but also in the comprehensive adjustment for MAP, as well as in the utilization of a large, ethnically homogeneous Japanese cohort.

Considering the characteristics of epidemiological studies, the mechanisms underlying the association between twin pregnancies and EO- and LO-HDP remain unclear. However, several fundamental studies have been conducted on this topic. The pathophysiology of PE differs between the EO- and LO-. The main cause of EO-PE is reported to be placental insufficiency, with soluble fms-like tyrosine kinase-1 (sFlt-1) and placental growth factor (PlGF) reported as markers. In pregnant women with EO-PE, the sFlt-1/PlGF ratio was significantly higher than that in pregnant women with LO-PE [44]. In twin pregnancies, as in singleton pregnancies, the sFlt-1/PlGF ratio is significantly higher in EO- than in LO-PE [45]. Additionally, in the comparison of non-PE groups between singleton and twin pregnancies, the sFlt-1/PlGF ratio was higher in twin pregnancies [46]. These reports imply a greater risk of placental insufficiency in twin pregnancies than in singleton pregnancies. Furthermore, the study findings demonstrated that the point estimate of the OR for the association between MD- and EO-HDP was higher than that for DD-twin pregnancies. This result is in agreement with previous studies reporting a higher sFlt-1/PlGF ratio in MD- than in DD-twin pregnancies [47], positioning our findings within the mechanistic hypothesis that increased placental load and dysfunction—particularly in MD-twin pregnancies—may underlie the elevated risk of EO-HDP. On the other hand, non-placental factors are also known to cause LO-PE through endothelial dysfunction [48]. Procopciuc et al. reported gene variants of AT1 (angiotensin II receptor type 1), a component of the RAS (renin–angiotensin system), in pregnant women with LO-PE [49]. No study has investigated the association between twin pregnancies and AT1. However, there have been reports on the association between twin pregnancies and the levels of ANG 1-7 (angiotensin 1-7) and ANG II (angiotensin II). Although both are factors of RAS, ANG 1-7 is associated with blood pressure reduction, whereas ANG II is associated with blood pressure elevation. Pawel et al. reported that the ratio of ANG 1-7 to ANG II in the third trimester was significantly higher in DD- than in MD-twin pregnancies [50]. This finding is consistent with the present study results, in which the OR for the association between MD-twin pregnancies and late-onset HDP was higher than that for DD-twin pregnancies, potentially reflecting differences in RAS-related vascular regulation and indicating that MD-twin pregnancies may be exposed to greater hemodynamic stress. Moreover, MAP levels were higher in twin pregnancies than in singleton pregnancies, and MAP levels were higher in MD- than in DD-twin pregnancies [18]. The difference between this study and that of previous studies was that the main outcomes were blood pressure levels and the timing of HDP onset. Furthermore, previous studies have reported that a high MAP level during early gestation is a risk factor for PE [51]. These findings support the hypothesis that twin pregnancies are at a higher risk of developing HDP than singleton pregnancies, and MD pregnancies have a higher incidence of PE than DD-twin pregnancies. Furthermore, the association between twin pregnancies and HDP persisted despite adjusting for MAP levels during early gestation. This suggests the presence of underlying mechanisms between twin pregnancies and HDP, which cannot be explained by MAP levels during early gestation. In twin pregnancies, maternal cardiovascular load is greater compared to singleton pregnancies, and MAP levels during pregnancy alone might be insufficient to adequately reflect hemodynamic changes such as cardiac output [52]. To elucidate these mechanisms, further investigation of longitudinal changes in relevant biomarkers, such as the sFlt-1/PlGF ratio and components of the RAS in twin pregnancies, is warranted.

The strength of this study is its large sample size, with participants recruited from a wide range of regions across Japan. Therefore, this study accounted for various covariates, including maternal factors, such as conception method and MAP, and socioeconomic factors. However, this study has several limitations. First, the subtypes of HDP (gestational hypertension, PE, and superimposed PE), family history of hypertension, and family history of PE were not collected in the JECS. Therefore, this study may have had some residual confounding factors. Second, due to differences in the proportion of HDP and twin pregnancies across countries, the generalizability of the findings to other populations remains uncertain. A previous study stratified by ethnicity reported that non-Hispanic black women had higher odds of HDP (OR: 1.28), whereas Hispanic (OR: 0.84) and Asian (OR: 0.85) women had lower odds than non-Hispanic white women [53]. The proportion of twin pregnancies is higher in European countries, such as Denmark, Greece, and the Netherlands, than in Japan [29]. Finally, biomarkers, such as sFlt-1, PlGF, AT1, ANG 1-7, and ANG II, were not measured in the JECS.

Low-dose aspirin (LDA) and correction of modifiable risk factors may be effective in reducing the risk of HDP in twin pregnancies. A previous systematic review reported that LDA could reduce the risk of PE in twin pregnancies, similar to its effects in singleton pregnancies [54]. A recent study reported that LDA could reduce the risk of HDP in singleton pregnancies [55]. However, these results must be validated in twin pregnancies. Correcting active and passive smoking in women with twin pregnancies may help prevent further increases in the risk of HDP [31, 56]. It is necessary to explore the other modifiable factors involved in the development of HDP. Moreover, future studies, including the measurement of biomarkers (sFlt-1, PlGF, AT1, ANG 1-7, and ANG II), are required to clarify the pathophysiology of the association between twin pregnancies and HDP. Although ambulatory blood pressure monitoring (ABPM) was not conducted in the JECS, it is considered useful for detecting white-coat hypertension and masked hypertension even in pregnant women, both of which are known to affect perinatal outcomes, including HDP [57, 58]. Furthermore, while morning blood pressure is recognized as an important risk factor for cardiovascular and cerebrovascular diseases [59, 60], research in pregnant populations remains limited [61]. In the future, the incorporation of ABPM—including assessment of morning blood pressure—in twin pregnancies, which carry a high risk of developing HDP, may contribute to more appropriate management and improved maternal and neonatal outcomes.

### Perspective of Asia

Moreover, the prevalence and risk factors for HDP and twin pregnancies in Asia may differ from those in other ethnic groups due to variations in genetic and socioeconomic backgrounds. This study contributes to the refinement of clinical guidelines in the Asian context by providing evidence derived from an ethnically homogeneous Japanese population. Future studies incorporating biomarkers and ABPM are warranted to develop prevention and management strategies tailored specifically to the Asian population.

In conclusion, both DD- and MD-twin pregnancies are risk factors for the development of EO- and LO-HDP.

## Supplementary information

Supplementary Information

Supplementary Table 1

Supplementary Fig. 1
