# Supplementary material for: Twin pregnancies are risk factors for both early- and late-onset hypertensive disorders of pregnancy: the Japan Environment and Children’s study
Source: Hypertens Res. 2026 Jan 9;49(4):1170–81. doi: 10.1038/s41440-025-02502-7 (PMC13050644; doi:10.1038/s41440-025-02502-7)
Supplement: Supplementary file 1 — Supplementary Information [file 41440_2025_2502_MOESM1_ESM.docx]

**SUPPLEMENTARY MATERIAL**

**Twin Pregnancies are Risk Factors for both Early- and Late-Onset Hypertensive Disorders of Pregnancy: The Japan Environment and Children’s Study**

Kazuma Tagami^a^, Noriyuki Iwama^a,b^, Hirotaka Hamada^a^, Hasumi Tomita^a^, Natsumi Kumagai^a^, Hongxin Wang^a^, Seiya Izumi^a^,Zen Watanabe^a^, Mami Ishikuro^c,d^, Taku Obara^c,d^, Hirohito Metoki^e,f^, Yuichiro Miura^g^, Chiharu Ota^g,h^, Shinichi Kuriyama^c,d,j^, Takahiro Arima^g^, Nobuo Yaegashi^g^, Masatoshi Saito^a,k^, and The Japan Environment and Children’s Study Group

^a^Department of Obstetrics and Gynecology, Tohoku University Graduate School of Medicine, 1-1, Seiryomachi, Sendai 980-8574, Miyagi, Japan

^b^Center for Maternal and Perinatal Medicine, Tohoku University Hospital, 1-1, Seiryomachi, Sendai, 980-8574, Miyagi, Japan

^c^Division of Molecular Epidemiology, Department of Preventive Medicine and Epidemiology, Tohoku Medical Megabank Organization, Tohoku University, 2-1, Seiryomachi, Sendai 980-8573, Miyagi, Japan

^d^Division of Molecular Epidemiology, Tohoku University Graduate School of Medicine, 2-1, Seiryomachi, Sendai 980-8575, Miyagi, Japan

^e^Division of Public Health, Hygiene and Epidemiology, Tohoku Medical Pharmaceutical University, 1-15-1 Fukumuro, Sendai 983-8536, Miyagi, Japan

^f^Tohoku Medical Megabank Organization, Tohoku University, Sendai, Miyagi, Japan

^g^Environment and Genome Research Center, Tohoku University Graduate School of Medicine, 2-1, Seiryomachi, Sendai 980-8575, Miyagi, Japan

^h^Department of Paediatrics, Tohoku University Graduate School of Medicine, 1-1, Seiryomachi, Sendai 980-8574, Miyagi, Japan

^i^Department of Obstetrics and Gynecology, Ehime University Graduate School of Medicine, Toon, Ehime 791-0295, Japan

^j^International Research Institute of Disaster Science, Tohoku University, 468-1, Aramaki, Sendai 980-8572, Miyagi, Japan

^k^Department of Maternal and Fetal Therapeutics, Tohoku University Graduate School of Medicine, 1-1, Seiryomachi, Sendai 980-8574, Miyagi, Japan

**Short running title:** Twin pregnancies and HDP

**Corresponding Author:**

Noriyuki Iwama, MD, PhD

Center for Maternal and Perinatal Medicine, Tohoku University Hospital, 1-1, Seiryomachi, Sendai 980-8574, Miyagi, Japan

Phone: +81-22-717-7251; Fax: +81-22-717-7258

Email: [noriyuki.iwama.a3@tohoku.ac.jp](mailto:noriyuki.iwama@med.tohoku.ac.jp)

**1. Differences in characteristics between study participants and pregnant women who were excluded due to missing data**

Supplementary Table 1 presented the differences in characteristics between the study participants and pregnant women who were excluded due to missing data on the history of chronic hypertension and the diagnosis of hypertensive disorders of pregnancy (HDP). Compared to study participants, pregnant women who were excluded were differ in maternal birth weight, pre-pregnancy body mass index, parity, conception method, mean arterial pressure level during early gestation, the history of mental disorders, smoking status, alcohol drinking status, highest maternal education level, annual household income, marital status, gestational diabetes mellitus, gestational weeks at delivery, and regions where Regional Centres exist.

**2. The differences in the risk of EO- and LO-HDP between DD- and MD-twin pregnancies.**

Supplementary Figure 1 also shows the differences in the risk of EO- and LO-HDP between DD- and MD-twin pregnancies. Although no statistically significant differences were observed, MD-twin pregnancies tended to have a higher odds of both EO- and LO-HDP compared with DD-twin pregnancies in model 2 (aOR: 1.44, 95% CI: 0.93–2.24 for EO-HDP, and aOR: 1.30, 95% CI: 0.90–1.88 for LO-HDP, respectively) and model 3 (aOR: 1.32, 95% CI: 0.86–2.05 for EO-HDP, and aOR: 1.24, 95% CI: 0.86–1.80 for LO-HDP, respectively).

**3. Appendix**

Members of the JECS Group as of 2025: Michihiro Kamijima (Principal Investigator, Nagoya City University, Nagoya, Japan), Shin Yamazaki (National Institute for Environmental Studies, Tsukuba, Japan), Maki Fukami (National Center for Child Health and Development, Tokyo, Japan), Reiko Kishi (Hokkaido University, Sapporo, Japan), Chiharu Ota (Tohoku University, Sendai, Japan), Koichi Hashimoto (Fukushima Medical University, Fukushima, Japan), Kenichi Sakurai (Chiba University, Chiba, Japan), Shuichi Ito (Yokohama City University, Yokohama, Japan), Ryoji Shinohara (University of Yamanashi, Chuo, Japan), Hidekuni Inadera (University of Toyama, Toyama, Japan), Takeo Nakayama (Kyoto University, Kyoto, Japan), Ryo Kawasaki (Osaka University, Suita, Japan), Yasuhiro Takeshima (Hyogo Medical University, Nishinomiya, Japan), Hideki Nagashima (Tottori University, Yonago, Japan), Narufumi Suganuma (Kochi University, Nankoku, Japan), Mayumi Tsuji (University of Occupational and Environmental Health, Japan), and Kimitoshi Nakamura (Kumamoto University, Kumamoto, Japan).

**Supplementary Table 1. Differences in characteristics between study participants and pregnant women who were excluded due to missing data**

**Supplementary Figure 1. The differences in the risk of EO- and LO-HDP between DD- and MD-twin pregnancies**
