# Supplementary material for: Twin pregnancies are risk factors for both early- and late-onset hypertensive disorders of pregnancy: the Japan Environment and Children’s study
Source: Hypertens Res. 2026 Jan 9;49(4):1170–81. doi: 10.1038/s41440-025-02502-7 (PMC13050644; doi:10.1038/s41440-025-02502-7)
Supplement: Supplementary file 2 — Supplementary Table 1 [file 41440_2025_2502_MOESM2_ESM.docx]

**Supplementary Table 1. Differences in characteristics between study participants and pregnant women who were excluded due to missing data**

| **Variables** | **Study participants (N=86,717)^1^** | **Pregnant women excluded due to missing data (N=1,765)^1^** | **p-value^2^** |
| --- | --- | --- | --- |
| **Maternal age, N (%)** | 30.8 (5.0) | 30.8 (5.2) | 0.8 |
| <25 years | 9,672 (11.2) | 64 (3.6) |  |
| 25－29.9 years | 25,449 (29.3) | 160 (9.1) |  |
| 30－34.9 years | 30,301 (34.9) | 208 (11.8) |  |
| 35－39.9 years | 18,146 (20.9) | 106 (6.0) |  |
| ≥40 years | 3,145 (3.6) | 29 (1.6) |  |
| Missing | 4 (0.0) | 1,198 (67.9) |  |
| **Maternal birth weight, N (%)** |  |  | <0.001 |
| <2,500 g | 3,814 (4.4) | 53 (3.0) |  |
| 2,500－3,999 g | 72,370 (83.5) | 743 (42.1) |  |
| ≥4,000 g | 1,780 (2.1) | 24 (1.4) |  |
| Missing | 8,753 (10.1) | 945 (53.5) |  |
| **Maternal height, cm** | 158.1 (5.3) | 158.0 (5.5) | 0.3 |
| **Pre-pregnancy BMI kg/m^2^, N (%)** | 21.1 (3.1) | 21.5 (3.9) | <0.001 |
| Underweight (<18.5 kg/m^2^) | 14,225 (16.4) | 295 (16.7) |  |
| Normal range (18.5－24.9kg/m^2^) | 63,907 (73.7) | 1,113 (63.1) |  |
| Overweight/Obesity (≥25.0 kg/m^2^) | 8,551 (9.9) | 235 (13.3) |  |
| Missing | 34 (0.0) | 122 (6.9) |  |
| **Parity, N (%)** |  |  | <0.001 |
| Primipara | 48,528 (56.0) | 865 (49.0) |  |
| Multipara | 36,089 (41.6) | 701 (39.7) |  |
| Missing | 2,100 (2.4) | 199 (11.3) |  |
| **Conception method, N (%)** |  |  | <0.001 |
| Spontaneous pregnancy | 80,430 (92.7) | 1,523 (86.3) |  |
| Non-ART | 3,284 (3.8) | 44 (2.5) |  |
| ART | 2,636 (3.0) | 40 (2.3) |  |
| Missing | 367 (0.4) | 158 (9.0) |  |
| **MAP level during early gestation, N (%)** | 79.1 (9.0) | 79.7 (9.6) | 0.03 |
| **SLE and/or APS** | 200 (0.2) | 0 (0.0) | 0.09 |
| **The history of kidney disease, N (%)** | 364 (0.4) | 0 (0.0) | 0.2 |
| **The history of mental disorders, N (%)** | 6,771 (7.8) | 59 (10.4) | 0.03 |
| **Type1 diabetes mellitus** | 60 (0.1) | 0 (0.0) | 1.0 |
| **Type2 diabetes mellitus** | 97 (0.1) | 2 (0.4) | 0.3 |
| **Smoking status, N (%)** |  |  | <0.001 |
| Never | 50,467 (58.2) | 322 (18.2) |  |
| Previously did, but quit before realizing current pregnancy | 20,039 (23.1) | 131 (7.4) |  |
| Previously did, but quit after realizing current pregnancy | 11,575 (13.3) | 73 (4.1) |  |
| Currently smoking | 4,005 (4.6) | 37 (2.1) |  |
| Missing | 631 (0.7) | 1,202 (68.1) |  |
| **Alcohol drinking status, N (%)** |  |  | <0.001 |
| Never | 29,698 (34.2) | 198 (11.2) |  |
| Quit drinking before | 47,874 (55.2) | 303 (17.2) |  |
| Continued drinking | 8,756 (10.1) | 61 (3.5) |  |
| Missing | 389 (0.4) | 1,203 (68.2) |  |
| **Highest maternal education level, N (%)** |  |  | <0.001 |
| <13 years | 30,446 (35.1) | 305 (17.3) |  |
| ≥13 years | 55,130 (63.6) | 533 (30.2) |  |
| Missing | 1,141 (1.3) | 927 (52.5) |  |
| **Annual household income (million, Japanese Yen), N (%)** |  |  | <0.001 |
| <4 | 31,841 (36.7) | 312 (17.7) |  |
| ≥4 | 39,374 (45.4) | 365 (20.7) |  |
| Missing | 15,502 (17.9) | 1,088 (61.6) |  |
| **Marital status, N (%)** |  |  | <0.001 |
| Married | 82,439 (95.1) | 517 (29.3) |  |
| Unmarried | 3,148 (3.6) | 36 (2.0) |  |
| Divorced or widowed | 746 (0.9) | 9 (0.5) |  |
| Missing | 384 (0.4) | 1,203 (68.2) |  |
| **New-onset HDP, N (%)** |  |  | 0.2 |
| EO-HDP | 445 (0.5) | 8 (0.8) |  |
| LO-HDP | 1,669 (1.9) | 25 (2.5) |  |
| **GDM, N (%)** | 2,268 (2.6) | 50 (4.2) | 0.001 |
| **Gestational weeks at delivery, weeks** | 39.2 (1.6) | 38.8 (2.1) | <0.001 |
| **Regions where Regional Centres exist, N (%)** |  |  | <0.001 |
| Hokkaido | 6,852 (7.9) | 180 (10.2) |  |
| Tohoku | 19,097 (22.0) | 153 (8.7) |  |
| Kanto | 10,266 (11.8) | 473 (26.8) |  |
| Chubu | 15,668 (18.1) | 272 (15.4) |  |
| Kinki | 14,695 (16.9) | 412 (23.3) |  |
| Chugoku | 2,622 (3.0) | 59 (3.3) |  |
| Shikoku | 5,975 (6.9) | 91 (5.2) |  |
| Kyushu-Okinawa | 11,542 (13.3) | 125 (7.1) |  |

^1^Continuous variables are expressed as means (SD). Categorical variables were expressed as N (%).

^2^Student t-test for continuous variables and Pearson’s Chi-squared test for categorical variables were conducted.

Abbreviations: APS, antiphospholipid antibody syndrome; ART, assisted reproductive technology; BMI, body mass index; GDM, gestational diabetes mellitus; EO, early onset; HDP, hypertensive disorders of pregnancy; LO, late onset; MAP, mean arterial pressure; SD, standard deviation; SLE, systemic lupus erythematosus.
