# Supplementary figures and images for: Twin pregnancies are risk factors for both early- and late-onset hypertensive disorders of pregnancy: the Japan Environment and Children’s study
Source: Hypertens Res. 2026 Jan 9;49(4):1170–81. doi: 10.1038/s41440-025-02502-7 (PMC13050644; doi:10.1038/s41440-025-02502-7)

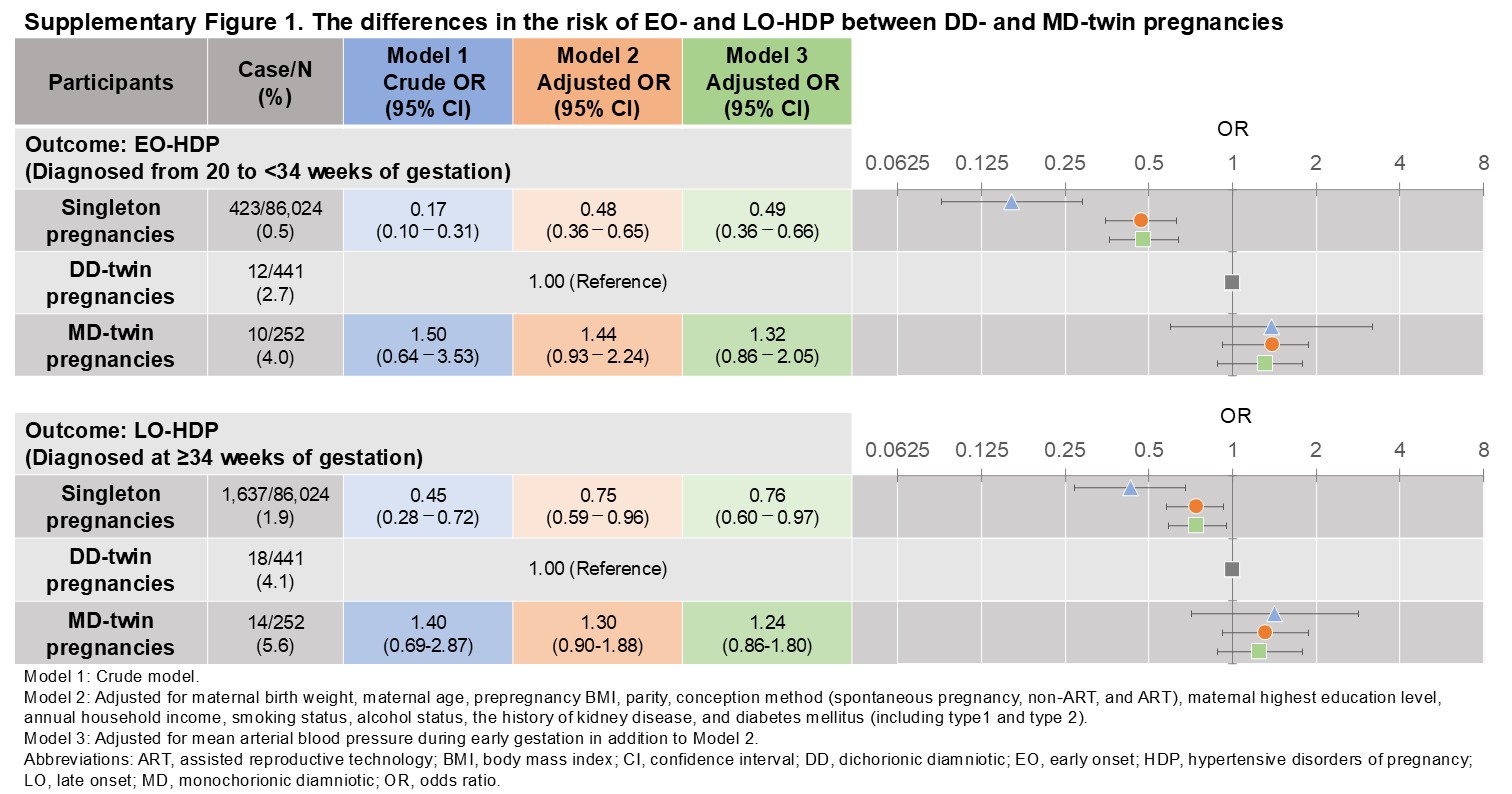

Supplement: Supplementary file 3 — Supplementary Fig. 1 [file 41440_2025_2502_MOESM3_ESM.jpg]
